# Hydrogen Sulfide Attenuates Particulate Matter-Induced Emphysema and Airway Inflammation Through Nrf2-Dependent Manner

**DOI:** 10.3389/fphar.2020.00029

**Published:** 2020-02-07

**Authors:** Guohua Jia, Siwang Yu, Wanlu Sun, Jin Yang, Ying Wang, Yongfen Qi, Yahong Chen

**Affiliations:** ^1^ Department of Pulmonary and Critical Care Medicine, Peking University Third Hospital, Beijing, China; ^2^ State Key Laboratory of Natural and Biomimetic Drugs, School of Pharmaceutical Sciences, Peking University, Beijing, China; ^3^ Key Laboratory of Molecular Cardiovascular Science, Ministry of Education, Peking University Health Science Center, Beijing, China

**Keywords:** chronic obstructive pulmonary disease, reactive oxygen species, air pollution, oxidative stress, emphysema, NLRP3, apoptosis

## Abstract

**Purpose:**

To investigate whether hydrogen sulfide provide protective effects on atmosphere particulate matter (PM)-induced emphysema and airway inflammation and its mechanism.

**Methods:**

Wild type C57BL/6 and Nrf2 knockout mice were exposed to PM (200 *µ*g per mouse). Hydrogen sulfide or propargylglycine were administered by intraperitoneal injection respectively 30 min before PM exposure, mice were anesthetized 29th day after administration. Mice emphysema, airway inflammation, and oxidative stress were evaluated, the expression of NLRP3, active caspase-1, and active caspase-3 were detected. Alveolar epithelial A549 cells line were transfected with control small interfering RNA (siRNA) or Nrf2 siRNA and then incubated with or without hydrogen sulfide for 30 min before exposed to fine particulate matter for 24 h, cell viability, terminal deoxynucleotidyl transferase deoxyuridine triphosphate nick-end labeling (TUNEL) assay, the secretion of interleukin (IL)-1*β*, ASC speck formation, the expression level of NLRP3, active caspase-1, and active caspase-3 were measured.

**Results:**

PM significantly increased mice emphysema and airway inflammation measured by mean linear intercept, alveolar destroy index and total cell, neutrophil counts, cytokines IL-6, tumor necrosis factor (TNF)-*α*, CXCL1, IL-1*β* in bronchoalveolar lavage fluid. PM-induced mice emphysema and airway inflammation was greatly attenuated by hydrogen sulfide, while propargylglycine aggravated that. PM-induced oxidative stress was reduced by hydrogen sulfide as evaluated by 8-OHdG concentrations in lung tissues. The expression of NLRP3, active caspase-1, and active caspase-3 enhanced by PM were also downregulated by hydrogen sulfide in mice lung. The protective effect of hydrogen sulfide on emphysema, airway inflammation, inhibiting oxidative stress, NLRP3 inflammasome formation, and anti-apoptosis was inhibited by Nrf2 knockout in mice. Similarly, hydrogen sulfide attenuated the secretion of IL-1*β*, NLRP3 expression, caspase-1 activation, ASC speck formation, and apoptosis caused by fine particulate matter exposure in A549 cells but not in Nrf2 silenced cells.

**Conclusion:**

Hydrogen sulfide played a protect role in PM-induced mice emphysema and airway inflammation by inhibiting NLRP3 inflammasome formation and apoptosis *via* Nrf2-dependent pathway.

## Introduction

Chronic obstructive pulmonary disease (COPD) is a disease characterized by airflow limitation and persisting respiratory symptoms which effected 8.6% people in China, accounting for 99.9 million people (Wang et al., 2018). Chronic bronchitis and emphysema are the main pathological changes, which is mainly caused by cigarette smoking and air pollution (Rabe and Watz, 2017). Atmosphere particulate matter (PM) increased COPD prevalence, exacerbations frequency and mortality, declined pulmonary function. (Li M. et al., 2016; Liu et al., 2017). Study showed that in PM-induced rat model, many pathogenesis that related to COPD were activated, including pulmonary inflammation measured by bronchoalveolar lavage fluid (BALF) inflammatory cell counts, increased inflammatory cytokine expression in BALF and serum, emphysematous changes, airway remodeling and mucus metaplasia (He et al., 2017).

Many mechanisms were related to PM-induced COPD model, in which oxidative stress, the inflammasome complex and cell apoptosis were of great concern (Zhang K. et al., 2018; Tien et al., 2019; Zhou et al., 2019). Nuclear factor erythroid 2 related factor 2 (Nrf2) is an important modulator regulating oxidative stress by enhancing downstream antioxidant enzymes or antioxidants expression like nicotinamide adenine dinucleotide phosphate (NADPH) quinone oxidoreductase 1 (NQO1), heme oxygenase-1, glutathione transferase, malondialdehyde, and superoxide dismutase (Lu et al., 2016). The Nrf2 related antioxidant system was impaired in tobacco smoke-induced COPD mice model and PM-induced COPD model, also activation of Nrf2 attenuated COPD and emphysema caused by cigarette smoke exposure (Sussan et al., 2009; Han et al., 2011; Zhang K. et al., 2018).

The inflammasome complex also play a key role in pathogenesis of many diseases, NACHT, LRR, and PYD domains-containing protein 3 (NLRP3) inflammasome was the member of inflammasome that involved in airway disease including COPD and emphysema pathogenesis. Recent studies showed that activation of NLRP3 inflammasome is important mechanism in PM-induced mice emphysema model (Birrell and Eltom, 2011; Uh et al., 2017). Also, PM was able to induce apoptosis of alveolar epithelial cell through multiple cell death pathways related to oxidative stress *in vivo* and *in vitro*, which contribute to the pathogenesis of pulmonary diseases caused by PM (Soberanes et al., 2006; Chuang et al., 2013; Deng et al., 2014; Huang et al., 2014; Peixoto et al., 2017). Therefore, finding effective methods or drugs resisting oxidative stress, inhibiting inflammasome formation and suppressing apoptosis to prevent and treat PM-induced lung damage like COPD or emphysema was of great significance.

We previous reported that hydrogen sulfide (H_2_S), a novel gaseous signal molecule, also severed as defense system in lung, was impaired in COPD patients (Chen et al., 2005; Sun et al., 2015). And H_2_S exerted significant protect role in defending against cigarette smoke or ozone exposure caused COPD/emphysema *via* the anti-oxidative stress, anti-apoptosis, anti-endoplasmic reticulum stress, anti-inflammatory function of H_2_S (Han et al., 2011; Li F. et al., 2016; Lin et al., 2017). Recent studies demonstrated that H_2_S inhibited reactive oxygen species (ROS) generation, NLRP3 inflammasome and apoptosis to improve endothelial dysfunction in spontaneously hypertensive rats, attenuate high glucose-induced human retinal pigment epithelial cell inflammation and attenuate pathogenesis of ozone-induced mice lung inflammation and emphysema (Li F. et al., 2016; Li et al., 2019; Wang et al., 2019). H_2_S also showed powerful protective effects on oxidative stress-dependent diseases though activation of Nrf2 pathway (Xie L. et al., 2016; Corsello et al., 2018). However, whether H_2_S protect against PM-caused emphysema, airway inflammation and whether H_2_S protect against PM-caused emphysema, airway inflammation through Nrf2-dependent manner was not known.

Therefore, we hypothesize that H_2_S protect against PM-induced emphysema and airway inflammation *via* antioxidative stress, inactivation of NLRP3 inflammasome and anti-apoptosis through Nrf2-dependent pathway.

## Materials and Methods

### Drugs and Reagents

Sodium hydrosulfide (NaHS) (70%, CAT# 161527) and propargylglycine (PPG) (98%, CAT# P7888) were purchased from Sigma Aldrich Chemical Co. (MO, USA). PM (urban particulate matter, standard reference material 1648a) was purchased from National Institute of Standards and Technology (MD, USA). Antibodies against ATCB (CAT# 4967S), NLRP3 (CAT# 15101) were purchased from Cell Signaling Technology (MA, USA), Nrf2 (CAT# ab62352), NQO1 (CAT# ab34173), and caspase-3 (CAT# ab13847) were purchased from Abcam (MA, USA), caspase-1 (CAT# AG-20B-0042) was purchased from Adipogen (CA, USA), cystathionine γ-lyase (CTH) (CAT# 12217-1-AP) was purchased from Proteintech (IL, USA), apoptosis-associated speck-like protein (ASC) (CAT# sc-271054) was purchased from Santa Cruz Biotechnology (TX, USA) and horseradish peroxidase (HRP)-conjugated secondary antibodies (CAT# A21010 and A21020) were purchased from Abbkine (Wuhan, China). Goat anti-mouse IgG H&L (Alexa Fluor 488) preadsorbed (CAT# ab150117) was purchased from Abcam (MA, USA). Enzyme-linked immunosorbent assay (ELISA) for mouse interleukin (IL)-1*β* (CAT# MLB00C), IL-6 (CAT# M6000B), CXCL1 (CAT# MKC00B) were purchased from R&D Systems (MN, USA), ELISA for mouse tumor necrosis factor (TNF)-*α* (CAT# ELM-TNF-*α*), 8-hydroxydeoxyguanosine (8-OHdG) (CAT# CEA660Ge) were purchased from Wuhan USCN Business Co. (Wuhan, China), ELISA for human IL-1*β* (CAT# ab100562) was purchased from Abcam (MA, USA). Wright-Giemsa dye, phosphate-buffered saline (PBS), reactive oxygen species assay kit [dichloro-dihydro-fluorescein diacetate (DCFH-DA)], and Hoechst 33258 were purchased from Solarbio Science & Technology Co (Beijing, China). *In Situ* Cell Death Detection Kit, fluorescein (CAT# 11684795910) was purchased from Roche (Basel, Switzerland). Lipofectamine 2000 transfection reagent, negative control siRNA (CAT# 4390843) and Nrf2 siRNA (CAT# 4392420) were purchased from Invitrogen (MA, USA). Cell counting kit-8 was purchased from Dojindo Laboratories (Shanghai, China).

### Animals and Treatments

The Nrf2 knockout mice were kindly provided by Dr. John D. Hayes (University of Dundee, Scotland, United Kingdom) and Dr. Masayuki Yamamoto (Tohoku University, Japan) (Itoh et al., 1997). The wide type (WT) C57BL/6 mice were purchased from Department of Laboratory Animal Science, Peking University Health Science Center. All mice were raised in a specific-pathogen-free (SPF) animal laboratory with constant temperature and humidity, feed by trained staffs. After 1 week of adaptive phase, 6–8 weeks old male mice were used for experiment. To establish PM-induced emphysema and airway inflammation model, 200 *µ*g PM (dilute with 50 *µ*l PBS) were delivered by intratracheal instillation, while control mice were administrated 50 *µ*l PBS (Li et al., 2017). To detect the protect effect of H_2_S on PM-induced emphysema and airway inflammation, NaHS (a exogenous donor of H_2_S, 50 *µ*M/kg) or PPG (a endogenous H_2_S blocker by inhibiting CTH, 50 *µ*M/kg) were given 30 min before PM administration by intraperitoneal injection. All mice were sacrificed at 29th day. Each group contains 12 mice, in which 6 were used to collect BALF and others were used for western blot, ELISA, and lung histology. Animal care and experimental protocols were approved by the Ethical Committee of Peking University Health Science Center (LA2019309).

### Histological Analysis and Quantification of Emphysema

After sacrificed, the left lungs of mice were removed and fixed with 4% paraformaldehyde for 24 h. Then, lung tissues were embedded in paraffin and cut into 4 *µ*m slices for hematoxylin and eosin (H&E) stain. The emphysema lesion was determined with alveolar spaces enlargement and alveolar walls destrection (Bracke et al., 2006). The enlargement of alveolar spaces was evaluated by mean linear intercept (Lm). Briefly, draw a 100 × 100 *µ*m grid over images of H&E stain using image analysis software (ImageJ 1.52K), the Lm was the average distance that the total length of each line of the grid divided by the number of alveolar intercepts (Thurlbeck, 1967). Destructive index (DI) was used to assess the destruction of alveolar walls. Draw a grid with 42 points on the image of H&E stains, the alveolar that each point fell into was counted as N (normal structure) or D (destroyed structure), the DI was calculated as D/(D+N) × 100% (Saetta et al., 1985).

### Collection of Bronchoalveolar Lavage Fluid and Inflammatory Cells Count

Six mice of each group were used to harvest BALF according to published protocol (Bracke et al., 2006). After stained with Wright-Giemsa dye, the total number of leukocytes and neutrophils in BALF were counted using a hemocytometer under optical microscope (Nikon, Japan).

### Measurement of Inflammatory Cytokines in Bronchoalveolar Lavage Fluid

The collected BALF were centrifuged at 4°C, 1500 rpm for 10 min, then the supernatant of BALF were stored at −80°C before use. The mouse airway inflammation was estimated by inflammatory cytokine including IL-6, CXCL1, TNF-*α*, and IL-1*β* in BALF, which were assessed by ELISA kits according to assay procedure.

### Measurement of Reactive Oxygen Species in Lung Tissues

Oxidative stress was evaluated with ROS generation and antioxidant enzymes in lung tissues. The ROS generation was detected by 8-OHdG using ELISA kit according to manufacturer instruction. Antioxidant enzymes expression including Nrf2, NQO1 were measured by western blot.

### PM_2.5_ Collection and Preparation

As previously described, a high volume air sampler with a pump flow rate of 1.13 m^3^/min was placed on the rooftop of the School of Public Health Building of Peking University in Beijing, China to collect PM_2.5_. The daily PM_2.5_ samples were collected on 90 mm Emfab filters (TX40HI20WW, part #7234, Pall Company, Beijing Office, Beijing, China) (Tripathi et al., 2018). To extract PM_2.5_, the filters were cut up into small pieces and placed in 90 ml sterile ultra-pure water, then sonicated for total 2 h with every 20 min gently shaking the beaker at constant temperature. Then the dissolved PM_2.5_ were filtered with gauze and freeze-dried for 24 consecutive hours to get dry PM_2.5_ sample. The extracted PM_2.5_ were weighted and dissolved in PBS at a concentration of 20 mg/ml for storage. The PM_2.5_ solution was completely mixed before every experiment.

### Cell Culture and Particulate Matter_2.5_ Exposure

Human alveolar epithelial A549 cell line were purchased from Medical Research Center of Peking University Third Hospital. A549 cells were cultured in Dulbecco's modified Eagle medium (DMEM) medium supplemented with 10% fetal bovine serum and incubated in a constant temperature incubator at 37°C with 5% CO_2_. Cells were treated with PM_2.5_ of different concentrations for 24 h to get the proper model for subsequent experiments. A549 cells were transfected with control siRNA or Nrf2 siRNA for 72 h and then incubated with or without 400 *µ*M NaHS for 30 min before PM_2.5_ exposure. After treatment, cellular supernatants were centrifuged and total proteins were collected for detection.

### Collection of Cellular Supernatant and Measurement of Inflammatory Cytokines

Cell culture supernatants were collected after A549 cells were exposed to PM_2.5_ for 24 h, centrifuged at 1,000×g for 20 min at 4°C condition, then cellular supernatants were stored at −80°C. The secretion IL-1*β* in cellular supernatants were detected by ELISA kit according to assay procedure.

### Measurement of Cell Viability

One-hundred microliters of suspension with 10^5^/ml A549 cells were cultured in 96-wall plate, after adhered to plate, cells were treated with PM_2.5_ for 24 h, then culture medium were removed and replaced with culture medium containing 10% cell counting kit-8. After incubation for 1 to 4 h, the 96-wall plate was measured using microplate reader at 450–490 nm to detect the cell viability.

### Measurement of Reactive Oxygen Species Generation in A549 Cells

A549 cells were cultured in 35 mm glass bottom cell culture dishes, after cells were treated with PM_2.5_ with or without H_2_S for 24 h, 10 *µ*M DCFH-DA probe was added and incubated in cell incubator for 20 min. After incubation, cells were washed with serum-free medium for three times and observed under fluorescence microscope in 30 min. To quantify the ROS generation, A549 cells were seeded in 96-well plate and processed as mentioned above, microplate reader with wavelengths of emission at 488 nm and excitation at 525 nm was used to detect fluorescence intensity.

### Terminal Deoxynucleotidyl Transferase Deoxyuridine Triphosphate Nick-End Labeling Assay

A549 cells were treated with or without H_2_S before PM_2.5_ exposure, then cells were fixed with 4% paraformaldehyde for 1 h at room temperature and rinsed with PBS. After that, cells were incubated with 0.1% Triton X-100 solution for 2 min on ice and rinsed twice with PBS. Next, 50 *µ*l TUNEL reaction mixture was added on cells and incubated in a humidified atmosphere for 1 h at 37°C in dark. After that, cells were rinsed with PBS and stained with Hoechst 33258. Finally, cells were analyzed under a fluorescence microscope.

### ASC Speck Formation

A549 cells were seeded in 35 mm glass bottom cell culture dishes, after stimulated with PM_2.5_ or H_2_S, cells were fixed with 4% paraformaldehyde and washed by PBS. Then, cells were permeabilized with 0.5% triton X-100 and blocked with 5% bovine serum albumin for 30 min, and then incubated with antibody anti-ASC (1:200 dilution) at 4°C condition overnight. After washed by PBS for three times, cells were incubated with Alexa Fluor 488 (1:1,000 dilution) for 30 min at room temperature. Finally, cells were rinsed with PBS and stained with Hoechst 33258. A fluorescence microscope was used to analyze the ASC speck formation.

### Western Blot

Lung tissues were mixed with radioimmunoprecipitation (RIPA) and phenylmethylsulfonyl (PMSF) to collect total proteins. The protein expression levels were quantified using Coomassie brilliant blue's method follow standard process. And then, equal amount proteins of each group were run on sodium dodecyl sulfate polyacrylamide gel electrophoresis (SDS-PAGE) gels and transferred to nitrocellulose membranes. After blocking with 5% skim milk, the membranes were incubated with primary antibodies against CTH (1:2,000 dilution), Nrf2 (1:2,000 dilution), NLRP3 (1:1,000 dilution), and caspase-1 (1:1,000 dilution), NQO1 (1:2,000 dilution), caspase-3 (1:1,000 dilution), ATCB (1:2,000 dilution) at 4°C overnight and then incubated with relevant HRP-conjugated secondary antibodies at room temperature for 1 h. The expression level of target protein was detected using Tanon 5200 Gel Imaging System (Tanon, Shanghai, China) according to standard manufacturer and analyzed with ImageJ 1.52k software.

### Statistical Analyses

Continuous numbers were expressed as mean ± SD. Differences between groups were analyzed using student's *t* test or one-way ANOVA when appropriate. All analysis were performed on SPSS 22.0 software (IBM, NY, USA) with a *p* value less than 0.05 (two tails) considered significant.

## Results

### H_2_S Attenuated Particulate Matter-Induced Emphysema in Wild-Type Mice

**Figure 1:**
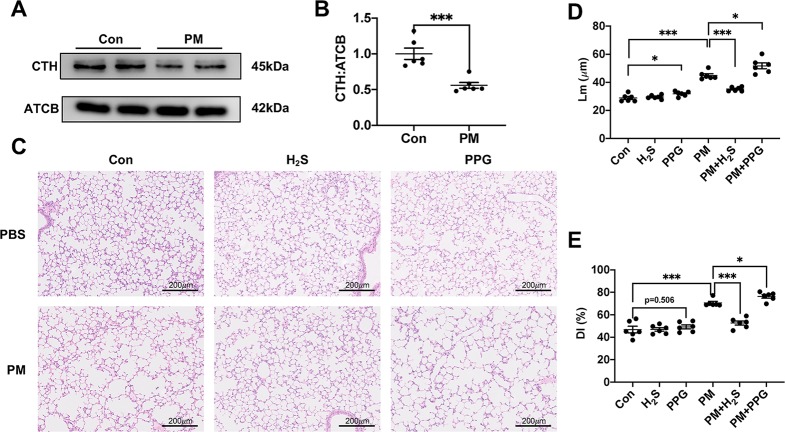
The effect of PM on H_2_S synthesis and effects of H_2_S on PM-induced emphysema in wild-type (WT) mice lung tissues. **(A**, **B)** The change of cystathionine γ-lyase (CTH) expression after PM exposure. **(C)** Representative images of hematoxylin and eosin (H&E) stain in control, H_2_S, PPG, PM, PM+H_2_S, PM+PPG group respectively (×100). **(D)** Changes in Lm of lung sections in each group. **(E)** Changes in DI of lung sections in each group. Results are expressed as mean ± SD; n = 6 in each group. *p< 0.05, ***p < 0.001 between groups. CTH, cystathionine γ-lyase; PM, particulate matter; H_2_S, hydrogen sulfide; PPG, propargylglycine; Lm, mean linear intercept; DI, destructive index.

After treatment with PM, the expression level of CTH was down-regulated in mice lungs (**Figures 1A, B**). To further investigate the role of H_2_S in PM-induced emphysema, we employed H_2_S donor NaHS and H_2_S inhibitor PPG before PM-exposure. As expected, NaHS successfully prevented PM-induced emphysema in WT mice measured by Lm and DI, while PPG aggregated that (**Figures 1C–E**).

### H_2_S Reduced Particulate Matter-Induced Airway Inflammation in Wild-Type Mice

**Figure 2:**
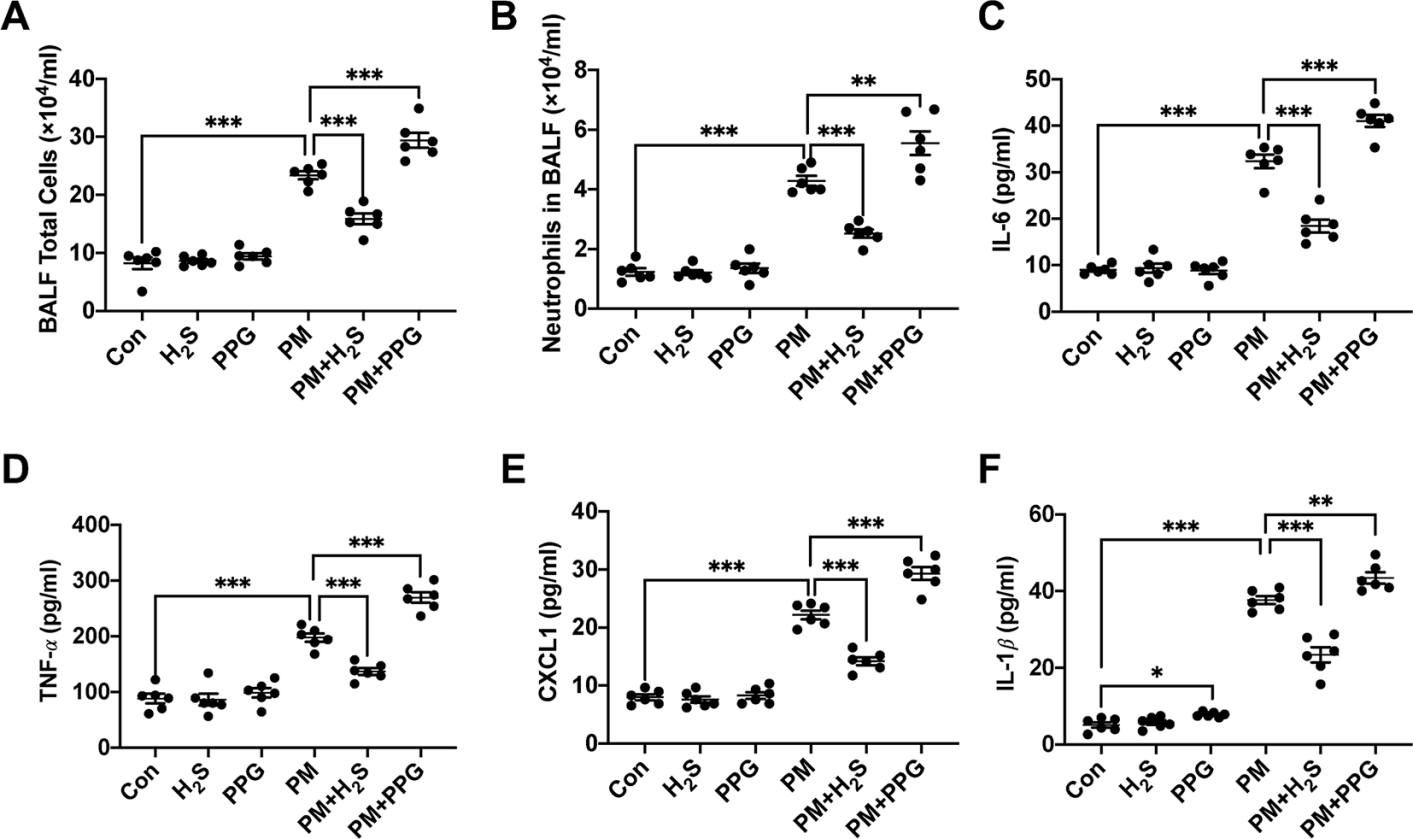
Effects of H_2_S on particulate matter (PM)-induced airway inflammation in wild-type (WT) mice lung. **(A**, **B)** Changes of total cells and neutrophilic leukocyte count in bronchoalveolar lavage fluid (BALF) in control, H_2_S, PPG, PM, PM+H_2_S, PM+PPG group respectively. **(C)** Changes of interleukin (IL)-6 level in BALF in each group. **(D)** Changes of tumor necrosis factor (TNF)-*α* level in BALF in each group. **(E)** Changes of CXCL1 level in BALF in each group. **(F)** Changes of IL-1*β* level in BALF in each group. Results are expressed as mean ± SD; n = 6 in each group. *p< 0.05, **p < 0.01, ***p < 0.001 between groups. BALF, bronchoalveolar lavage fluid; H_2_S, hydrogen sulfide; PPG, propargylglycine; PM, particulate matter.

To verify whether H_2_S protected against PM-induced airway inflammation, we measured airway inflammation in PM exposed mice with or without H_2_S donor and inhibitor. The results showed that NaHS significantly reduced the enhanced airway inflammation including total and neutrophil cells number, IL-6, TNF-*α*, CXCL1, and IL-1*β* protein expression in BALF, on the contrary, PPG aggregated that (**Figures 2A–F**). Taken together, our results indicated that PM-suppressed the production of H_2_S in mice lung, and H_2_S showed protective efforts on PM-induced emphysema and airway inflammation.

### H_2_S Failed to Prevent Particulate Matter-Induced Emphysema in Nrf2−/− Mice

**Figure 3:**
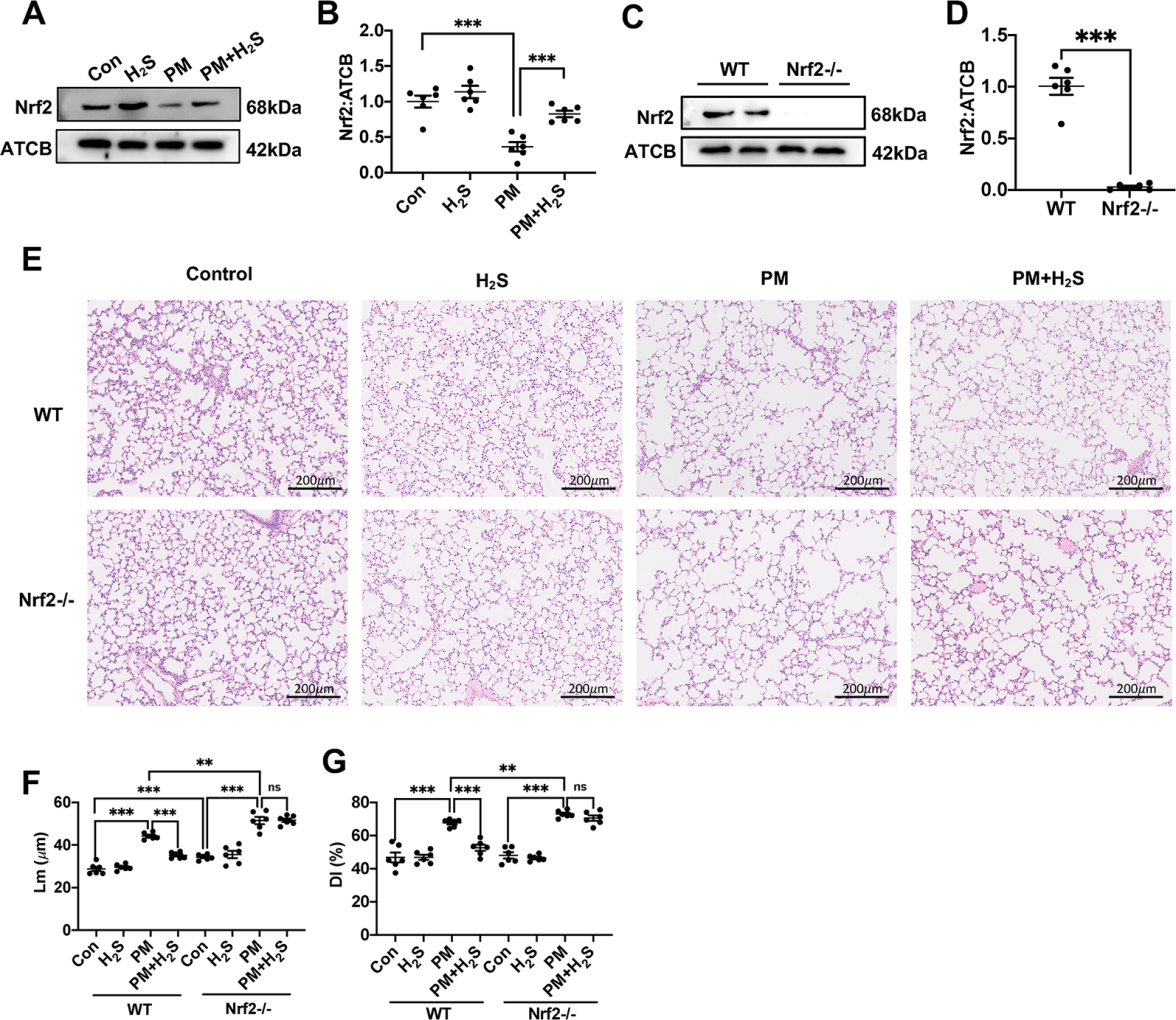
Effects of H_2_S on PM-induced emphysema in Nrf2−/− mice. **(A**, **B)** Changes in Nrf2 expression of control, H_2_S, PM, PM+H_2_S group respectively. **(C**, **D)** The Nrf2 expression level in WT and Nrf2−/− mice lung. **(E)** Representative images of hematoxylin and eosin (H&E) stain of control, H_2_S, PM, PM+H_2_S group in WT and Nrf2−/− mice respectively (×100). **(F)** Changes in Lm of lung sections in each group. **(G)** Changes in DI of lung sections in each group. Results are expressed as mean ± SD; n = 6 in each group. ns **p < 0.01, ***p < 0.001 between groups. Nrf2, nuclear factor erythroid 2 related factor 2; PM, particulate matter; H_2_S, hydrogen sulfide; WT, wide type; Lm, mean linear intercept; DI, destructive index.

The expression level of Nrf2 was significantly decreased in WT mice lung and NaHS reversed that (**Figures 3A, B**). Then, we used Nrf2 knockout mice to detect the role of Nrf2 in protective effects of H_2_S on PM-induced emphysema. The Nrf2 knockout efficiency was verified by the protein expression level of Nrf2 in lung tissues (**Figures 3C, D**). In contrast to WT mice, H_2_S failed to prevent PM-induced emphysema in Nrf2−/− mice (**Figures 3E–G**).

### H_2_S Failed to Alleviate Particulate Matter-Induced Airway Inflammation in Nrf2−/− Mice

**Figure 4:**
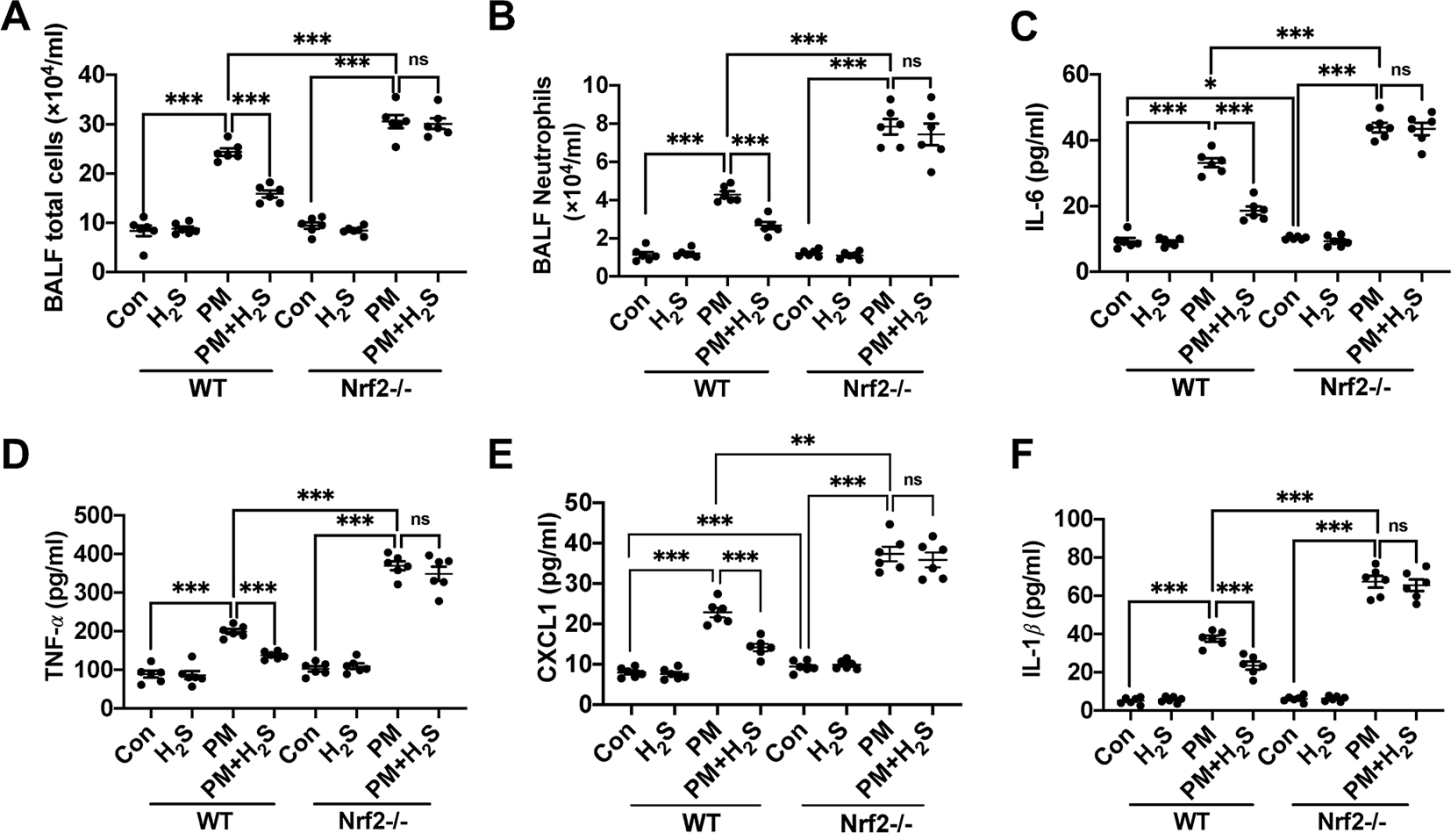
Effects of H_2_S on PM-induced airway inflammation in Nrf2−/− mice lung. **(A**, **B)** Changes of total cells and neutrophilic leukocyte count in BALF of control, H_2_S, PM, PM+H_2_S group in WT and Nrf2−/− mice. **(C)** Changes of BALF interleukin (IL)-6 expression level in each group in WT and Nrf2−/− mice. **(D)** Changes of BALF tumor necrosis factor (TNF)-*α* expression level in each group in WT and Nrf2−/− mice. **(E)** Changes of BALF CXCL1 expression level in each group in WT and Nrf2−/− mice. **(F)** Changes of BALF IL-1*β* expression level in each group in WT and Nrf2−/− mice. Results are expressed as mean ± SD; n = 6 in each group. ns *p* > 0.05, *p< 0.05, **p < 0.01, ***p < 0.001 between groups. BALF, bronchoalveolar lavage fluid; H_2_S, hydrogen sulfide; PM, particulate matter; WT, wide type.

Similarly, we tested whether H_2_S showed same protective role against PM-induced airway inflammation as WT mice in Nrf2−/− mice. We found that H_2_S showed no protective efforts on PM-induced airway inflammation in Nrf2−/− mice (**Figures 4A–F**). The above results showed that H_2_S protected against PM-induced emphysema and airway inflammation *via* the activation of Nrf2 pathway.

### H_2_S Prevented the Reactive Oxygen Species Generation, NLRP3 Inflammasome Formation and Apoptosis in Wild-Type Mice but Not in Nrf2−/− Mice

**Figure 5:**
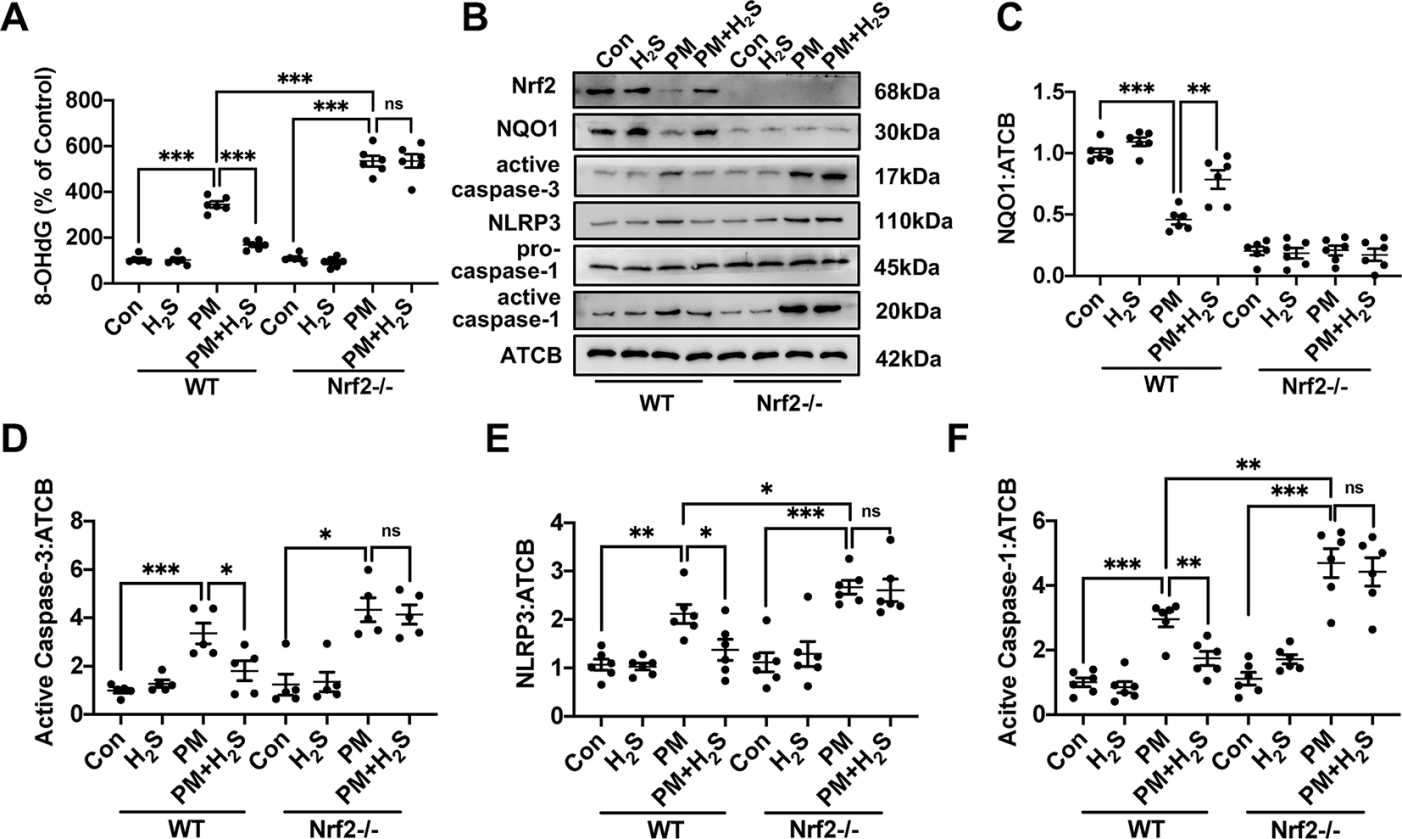
Effects of H_2_S on reactive oxygen species (ROS) generation, NLRP3 inflammasome formation and apoptosis after PM exposure in WT and Nrf2−/− mice lung tissues. **(A)** Changes of ROS generation in control, H_2_S, PM, PM+H_2_S group in WT and Nrf2−/− mice lung. **(B**–**F)** Changes of Nrf2, NQO1, active caspase-3, NLRP3, and active caspase-1 (p20) expression level in control, H_2_S, PM, PM+H_2_S group in WT and Nrf2−/− mice lung respectively. Results are expressed as mean ± SD; n = 5–6 in each group. ns *p* > 0.05, *p< 0.05, **p < 0.01, ***p < 0.001 between groups. 8-OhdG, 8-hydroxydeoxyguanosine; H_2_S, hydrogen sulfide; PM, particulate matter; WT, wide type; Nrf2, Nrf2, nuclear factor erythroid 2 related factor 2; NQO1, NADPH quinone oxidoreductase 1; NLRP3: NACHT, LRR, and PYD domains-containing protein.

To further explore the mechanism how H_2_S protect against PM-caused emphysema and airway inflammation, we detected the change of ROS generation, NLRP3 inflammasome formation, and apoptosis. We found that PM significantly increased 8-OHdG concentration and H_2_S prevented this phenomenon only in WT mice (**Figure 5A**). As could be expected, H_2_S enhanced the down-regulated Nrf2 and NQO1 induced by PM exposure in WT mice, but showed no change in Nrf2−/− mice. PM exposure also induced the activation of NLRP3 inflammasome and activated caspase-1 (p20), and H_2_S decrease the enhanced change of NLRP3 inflammasome and its downstream proteins only in WT mice. Similarly, cell apoptosis induced by PM was also inhibited by H_2_S in WT mice but not in Nrf2−/− mice (**Figures 5B–F**).

### H_2_S Prevented Particulate Matter_2.5_-Induced Reactive Oxygen Species Generation, NLRP3 Inflammasome Formation, and Apoptosis in A549 Cells but Not in Nrf2 Silenced A549 Cells

**Figure 6:**
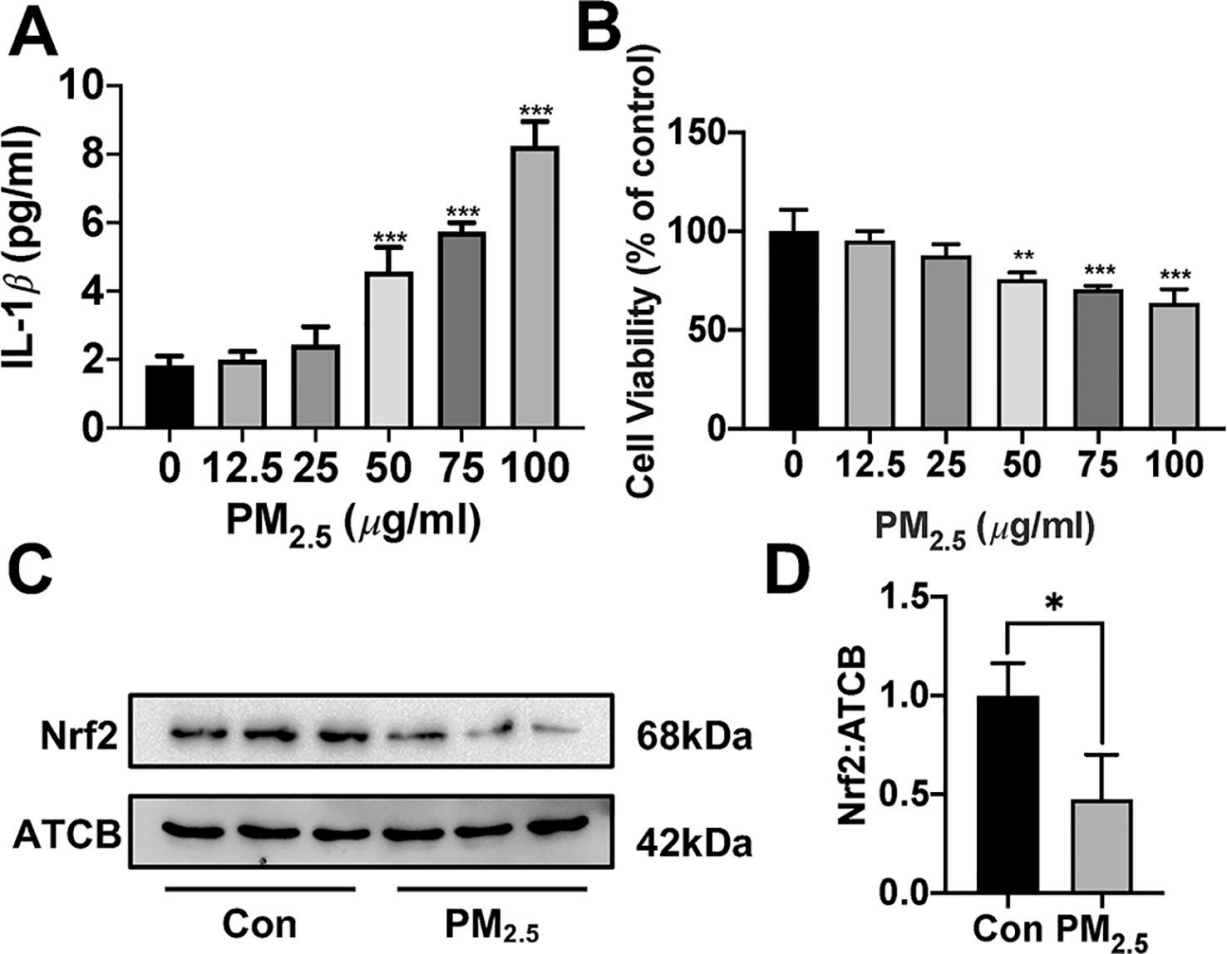
Effects of PM_2.5_ on interleukin (IL)-1*β* secretion, cell viability, and Nrf2 expression in A549 cells. **(A)** A549 cells were treated with PM_2.5_ of different concentrations for 24 h, the IL-1*β* in cell culture supernatant was detected to represent the IL-1*β* secretion in A549 cell of different groups (n = 5). **(B)** Cell viability were measured after treatment with PM_2.5_ of different concentrations for 24 h (n = 5). **(C, D)** A549 cell were treated with 50 *µ*g/ml PM_2.5_ for 24 h, the Nrf2 expression level were detected in control and PM_2.5_ group (n = 3). Results are expressed as mean ± SD. *p< 0.05, **p < 0.01, ***p < 0.001 between groups. PM_2.5_, fine particulate matter; Nrf2, nuclear factor erythroid 2 related factor 2.

**Figure 7:**
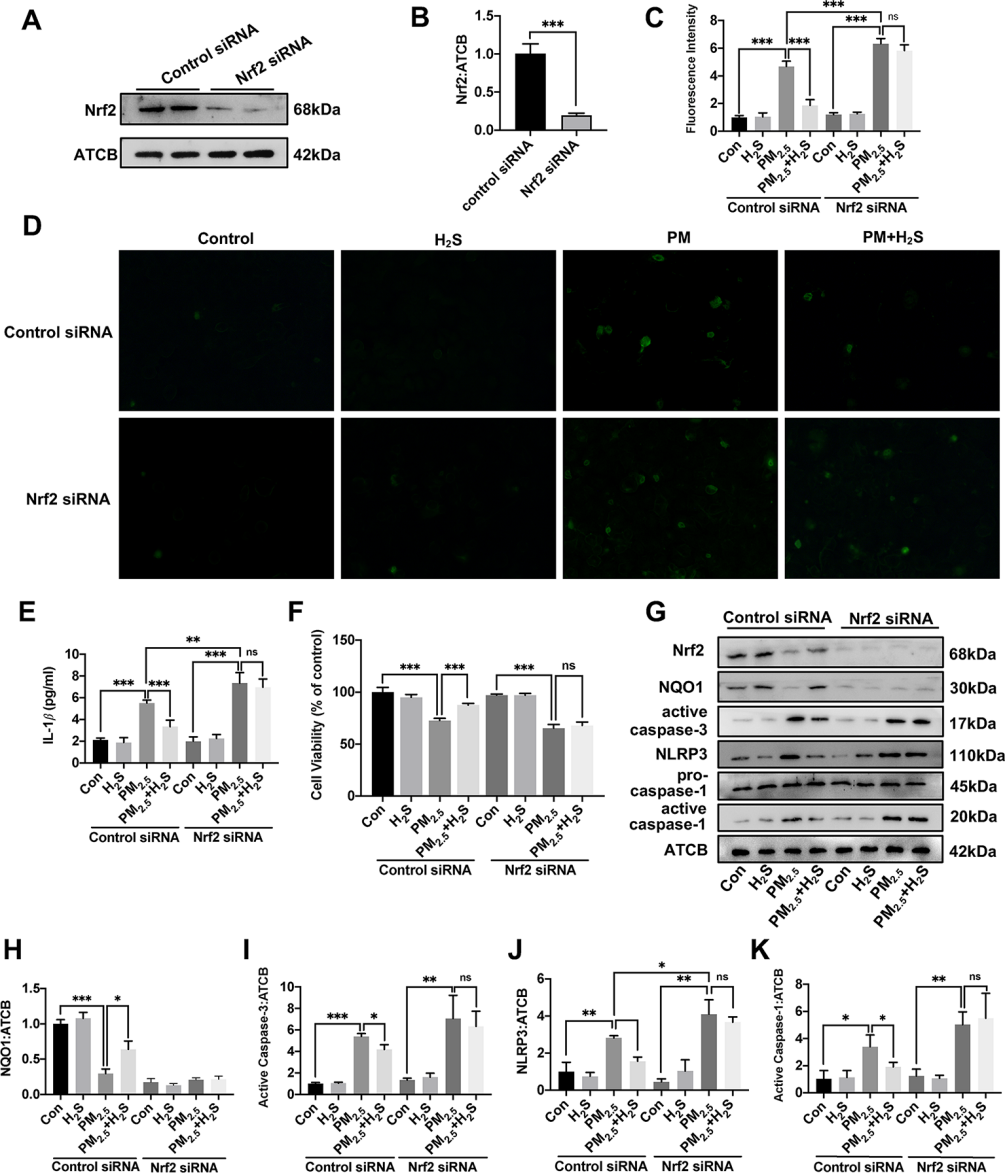
Effects of H_2_S on reactive oxygen species (ROS) generation, NLRP3 inflammasome formation, and cell apoptosis in A549 cells after PM_2.5_ stimulation. **(A**, **B)** The knocking-down efficiency of Nrf2 small interfering RNA (siRNA) in A549 cells. **(C)** Effects of H_2_S on ROS generation in control, H_2_S, PM_2.5_, PM_2.5_+H_2_S group in control siRNA, or Nrf2 siRNA transfected A549 cells respectively (n = 5). **(D)** Representative fluorescent images of ROS (green) in each group (×200). **(E)** Effects of H_2_S on the secretion of interleukin (IL)-1*β* in A549 cells after PM_2.5_ exposure. **(F)** Effects of H_2_S on cell viability in each group respectively. **(G**–**K)** Effects of H_2_S on Nrf2, NQO1, active caspase-3, NLRP3, active caspase-1 (p20) expression in A549 cells after PM_2.5_ exposure. Results are expressed as mean ± SD; three independent experiments were done in each group. ns *p* > 0.05, *p< 0.05, **p < 0.01, ***p < 0.001 between groups. Nrf2, nuclear factor erythroid 2 related factor 2; H_2_S, hydrogen sulfide; PM_2.5_, fine particulate matter; NLRP3, NACHT, LRR, and PYD domains-containing protein 3.

**Figure 8:**
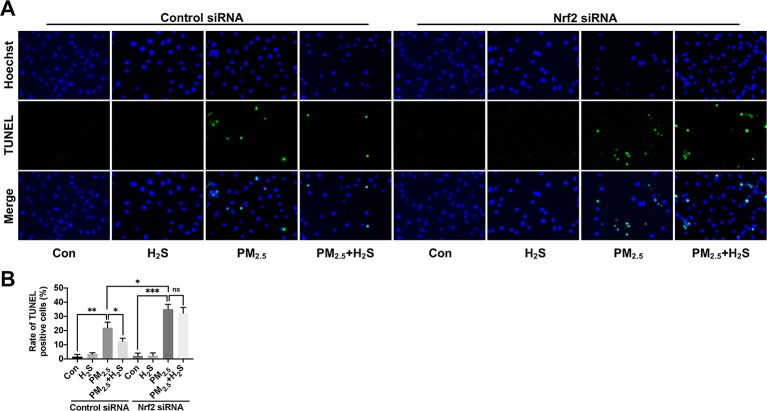
Effect of H_2_S on A549 cells apoptosis after PM_2.5_ exposure using terminal deoxynucleotidyl transferase deoxyuridine triphosphate nick-end labeling (TUNEL) assay. **(A)** Representative fluorescent images of Hoechst (blue), TUNEL (green), and merge in control, H_2_S, PM_2.5_, PM_2.5_+H_2_S group in control small interfering RNA (siRNA) or Nrf2 siRNA transfected A549 cells respectively (×400). **(B)** Percentage of TUNEL positive cells in each group respectively. Results are expressed as mean ± SD; three independent experiments were done in each group. ns *p* > 0.05, *p< 0.05, **p < 0.01, ***p < 0.001 between groups. Nrf2, nuclear factor erythroid 2 related factor 2; H_2_S, hydrogen sulfide; PM_2.5_, fine particulate matter.

**Figure 9:**
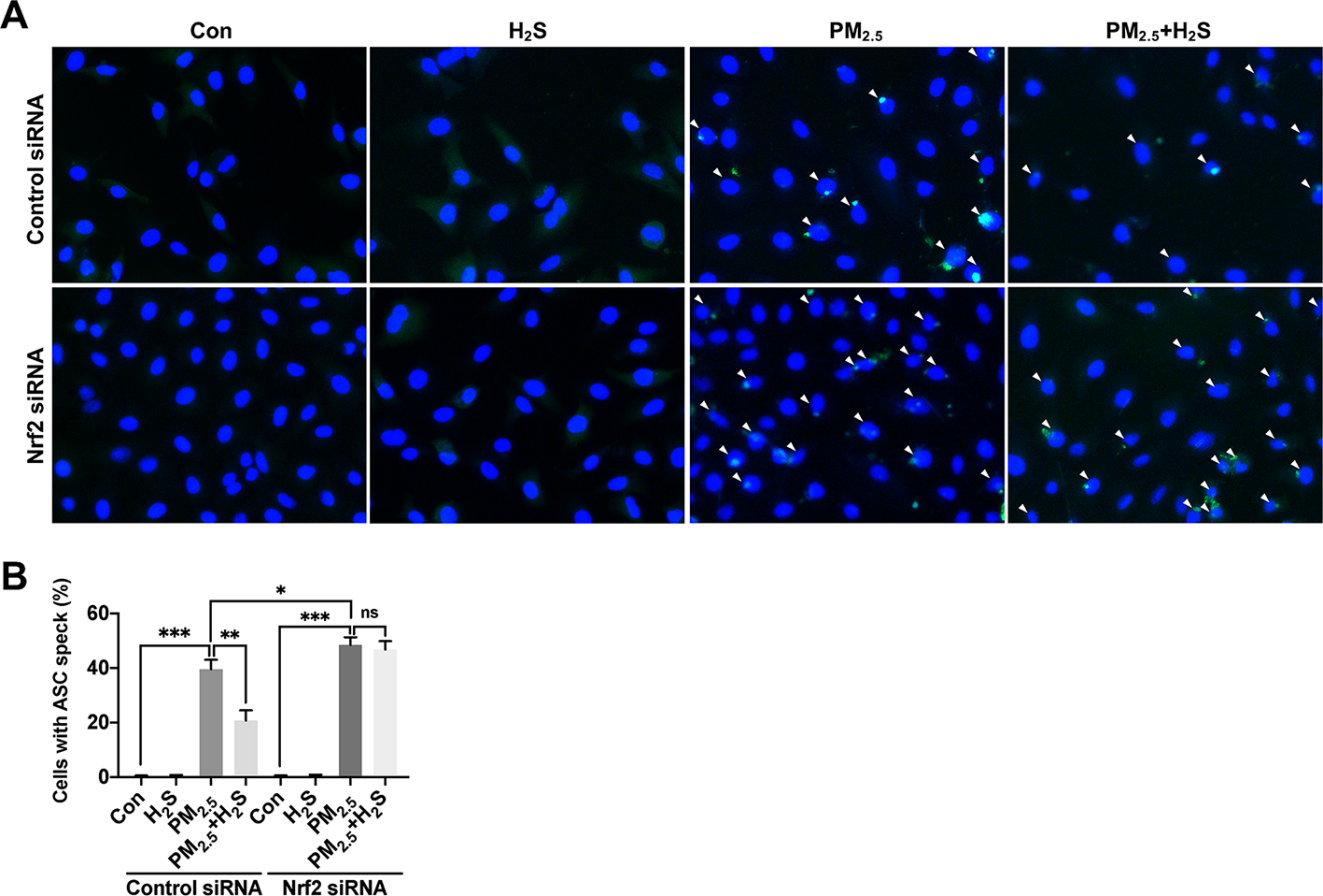
Effect of H_2_S on ASC speck formation in A549 cells after PM_2.5_ exposure. **(A)** Representative fluorescent images of Hoechst (blue) and ASC (green) merge in control, H_2_S, PM_2.5_, PM_2.5_+H_2_S group in control small interfering RNA (siRNA) or Nrf2 siRNA transfected A549 cells respectively (×400). **(B)** Percentage of ASC speck positive cells (white arrow) in each group respectively. Results are expressed as mean ± SD; three independent experiments were done in each group. ns *p* > 0.05, *p< 0.05, **p < 0.01, ***p < 0.001 between groups. Nrf2, nuclear factor erythroid 2 related factor 2; H_2_S, hydrogen sulfide; PM_2.5_, fine particulate matter; ASC, apoptosis-associated speck-like protein.

Since about 61% proportion of PM were distributed at alveolar walls in COPD patients, and PM_2.5_ were main components that got deeply into lung alveoli, so we used PM_2.5_ to stimulate alveolar epithelial A549 cell line to further confirm our hypothesis (Ling et al., 2011). As results shown, PM_2.5_ induced A549 cell apoptosis, enhanced the IL-1*β* secretion and down-regulated Nrf2 expression with PM_2.5_ concentration of 50 *µ*g/ml (**Figure 6**). To confirm whether H_2_S exerted protective effects and the role of Nrf2 in that, we transfected A549 with control siRNA or Nrf2 siRNA (**Figures 7A, B**). We found that H_2_S prevented the ROS generation, NLRP3 inflammasome formation, and apoptosis induced by PM_2.5_ in A549 cells, moreover, Nrf2 knocking-down blocked the protective effects of H_2_S (**Figures 7C–K**, **8**, and **9**).

## Discussion

In this research, we had many novel findings: 1) H_2_S attenuated PM-mediated emphysema and airway inflammation in mice; 2) H_2_S inhibited PM-caused emphysema and airway inflammation *via* Nrf2-dependent antioxidant stress pathway. 3) Protective effects of H_2_S on PM-induced emphysema and airway inflammation was associated with the antioxidant stress, inactivation of NLRP3 inflammasome, and anti-apoptosis role.

PM mainly resident in lung tissues including alveolar walls, macrophages, blood vessels, and airway walls of smokers and non-smokers, and was associated with COPD pathogenesis and COPD acute exacerbations (Ling et al., 2011; Li M. et al., 2016; Wang et al., 2018). Recent study showed that PM was able to induced emphysema and airway inflammation in mice, but the mechanism was not fully understood (Li et al., 2017). In this research, we found that CTH, the main enzyme products H_2_S in lung tissues, was impaired in PM-induced mice emphysema and airway inflammation model, which was consistent with our previous study reported that the protein level of CTH was impaired in smokers and COPD patients (Sun et al., 2015). Moreover, using PPG to inhibit endogenous H_2_S generation aggregated PM-caused lung injury, while the complement of exogenous H_2_S donor NaHS significantly attenuated the PM-induced lung damage. The results was in agreement with other studies that H_2_S defensed against cigarette smoke or ozone induced-COPD/emphysema, which further demonstrated that H_2_S was endogenous protection system *in vivo* and *in vitro* (Li F. et al., 2016; Lin et al., 2017). Though our study demonstrated that PM impaired endogenous H_2_S generation to cause lung damage, however, how PM decreased endogenous CTH expression level needs further study.

Oxidative stress is one of the most important mechanisms in COPD pathogenesis, and PM was also able to increase ROS generation to induce COPD (Huang et al., 2018; Zhao et al., 2019). Furthermore, targeting ROS treatment by N-acetylcysteine or taurine had showed significant effects in ameliorating PM-conducted adverse lung changes, which indicated that H_2_S may exert protective effects by antioxidant stress manner (Li et al., 2017; Liu et al., 2018). Consist with published studies, we also mentioned a great obviously down-regulated antioxidant related proteins and up-regulated ROS generation in our study, and H_2_S greatly reversed that (Wang et al., 2012). In spite of oxidation resistance efforts of H_2_S itself by increasing glutathione and thioredoxin synthesis, the antioxidant effort of H_2_S itself is limited, however, H_2_S exerted similar scavenging ROS function as N-acetylcysteine in PM-induced human lung endothelial barrier disruption, since H_2_S is the one of the Nrf2 agonist, the antioxidant stress role of H_2_S may due to the activation of Nrf2 (Wang et al., 2012; Xie Z. et al., 2016). Thus, we investigated whether H_2_S exerted protective effects by Nrf2-dependent manner. In PM-induced lung epithelial cells apoptosis model, Nrf2 was down-regulated after PM exposure (Wang et al., 2017). Similarly, the expression level of Nrf2 in mice and A549 cells was also down-regulated after PM exposure, while H_2_S prevented the decline of Nrf2 expression. To further confirm the function of Nrf2 antioxidant system in the protective effects of H_2_S, we used Nrf2 knockout mice model and Nrf2 silenced A549 cell model, however, the protective role of H_2_S was inhibited *in vivo* and *in vitro* after the blocking of Nrf2. Our results further indicated that the PM-caused lung damage were mainly ROS drove and H_2_S treated PM-caused emphysema and airway inflammation was *via* Nrf2/ROS pathway.

The inflammasome was involved in the pathogenesis of COPD and associated with COPD exacerbations (Lee et al., 2016). NLRP3 is one of the inflammasomes that mediate immune responses to inflammatory stimuli, the inflammasomes can be activated by a variety of activators like LPS, mitochondrial dysfunction, K+ efflux, *et al*., and ROS was the most common activator (Abais et al., 2015; He et al., 2016). The NLRP3 was also an important mechanism in cigarette smoking, ozone-induced mice COPD or emphysema (Lee et al., 2016; Li F. et al., 2016). In this study, the NLRP3 inflammasome also played an important part in PM-induced emphysema and airway inflammation *in vivo* and *in vitro* with the results that the IL-1*β* expression, NLRP3, and active caspase-1 (p20) was enhanced in mice and A549 cells after PM exposure. H_2_S showed inhibiting NLRP3 role in retinal pigment epithelial cell inflammation, colitis, and atherosclerosis (Qin et al., 2019; Wang et al., 2019; Yue et al., 2019). In this research, we also found that the NLRP3 formation can be inhibited by H_2_S *in vivo* and *in vitro*. Conversely, the inhibition NLRP3 along with the protection role of H_2_S was blocked by Nrf2 knockout mice and Nrf2 silenced A549 cells. These results suggested that the protective role of H_2_S was associated with the inactivation of NLRP3 inflammasome *via* Nrf2-dependent antioxidant stress manner.

Our previous study showed that H_2_S was able to inhibit cigarette smoke-induced apoptosis in rat lung and bronchial epithelial cells (Lin et al., 2017). Its also reported that the activation of Nrf2 by bixin protected against PM_2.5_-induced lung injury by alleviating oxidative stress, increasing proliferation and migration, decreasing apoptosis (Zhang H. et al., 2018; Liu et al., 2019). Thus, we investigate the effects of H_2_S/Nrf2 signaling on PM-conducted apoptosis. In our study, PM-induced apoptosis in mice lung and A549 cells were attenuated by H_2_S, moreover, while using Nrf2 knockout mice or Nrf2 silenced A549 cell model to arrest the Nrf2 expression, the anti-apoptosis of H_2_S was also blocked, which indicated that H_2_S mediated PM-induced apoptosis *via* the activation of Nrf2. These findings suggested that the Nrf2 activation mediated by H_2_S could be used to treat PM-related lung disease.

Our study had several advantages. The Nrf2 knockout mice and Nrf2 silenced cell used in our study allowed us to fully investigate the role of Nrf2 in the protective efforts of H_2_S. As only PM_2.5_ can get into bottom of the lung and deposit on alveoli, we used the PM_2.5_ instead of PM to stimulate alveolar epithelial cells, which was more likely to reveal the mechanism of emphysema pathogenesis in real world.

There are also some limitations in our research. There are many other mechanisms associated with PM-induced lung damage, further studies were needed to investigate whether H_2_S affect these pathways in PM-induced emphysema and airway inflammation, like Sirt1, autophagy, ageing, which had interaction both with H_2_S and Nrf2. The mechanism how H_2_S suppressed PM-induced cell apoptosis needs further study. Moreover, it's still unclear how PM downregulated H_2_S synthesize in lung. These remain to be research in future studies.

## Conclusion

In conclusion, our results demonstrated that H_2_S ameliorated PM-conducted lung emphysema and airways inflammation by scavenging ROS generation, inhibiting NLRP3 inflammasome formation, and anti-apoptosis *via* Nrf2 manner. H_2_S could be potential therapeutic measure preventing and treating air pollution induced lung injury.

## Data Availability Statement

The raw data supporting the conclusions of this article will be made available by the authors, without undue reservation, to any qualified researcher.

## Ethics Statement

The animal study was reviewed and approved by the Ethical Committee of Peking University Health Science Center.

## Author Contributions

GJ, SY, WS, YQ, JY, YW, and YC were responsible for the conception and design, analysis and interpretation of data, drafting the article or revising it critically for important intellectual content, and final approval of the version to be published, and all agree to be accountable for all aspects of the work in ensuring that questions related to the accuracy or integrity of any part of the work are appropriately investigated and resolved. All authors have read the manuscript and approve its submission.

## Conflict of Interest

The authors declare that the research was conducted in the absence of any commercial or financial relationships that could be construed as a potential conflict of interest.
